# Discordance Between Thoughts of Death and Suicidal Ideation Among Latinx Youth and Caregivers in the United States

**DOI:** 10.1002/jad.70143

**Published:** 2026-03-23

**Authors:** Lauren M. O’Reilly, Trey V. Dellucci, Matthew C. Aalsma, Natalie Guerrero

**Affiliations:** 1 Department of Psychiatry, Indiana University School of Medicine, Indianapolis, Indiana, USA; 2 Department of Pediatrics, Indiana University School of Medicine, Indianapolis, Indiana, USA

**Keywords:** adolescents, disagreement, discordance, dyads, suicidal ideation, thoughts of death

## Abstract

**Introduction:**

Despite increasing rates of suicidal thoughts and behavior among Latinx populations in the US, no prior research has examined Latinx youth and caregiver disagreement (discordance) in youth-reported thoughts of death or suicidal thoughts, as well as factors associated with discordance.

**Methods:**

Data were derived from youth aged 13–17 (M = 14.99 [SD = 1.52]) and caregivers (*n* = 127 dyads) who identified as Latinx/Hispanic living in a US Midwest city. The youth sample was mostly (61.7%) assigned female at birth; all youth identified as cisgender. Youth thoughts of death and suicidal thoughts were indexed with the Mood and Feelings Questionnaire. Predictors of discordance included demographics (e.g., age), youth psychopathology (e.g., internalizing and externalizing symptoms), caregiver anxiety, interpersonal factors (e.g., family conflict), and ethnic discrimination, identity, and socialization. Percent agreement and Cohen’s Kappa were used to describe discordance. Univariable logistic regression was used to examine predictors of discordance calculated in two ways: (1) youth endorsement/caregiver denial, and (2) youth denial/caregiver endorsement.

**Results:**

Among the final analytic sample of 120 dyads, agreement between caregiver and youth was minimal or weak (*κ* = 0.24–0.43). Caregiver and youth denial discordance were associated with nearly every predictor domain. For example, greater ethnic socialization was associated with reduced odds of caregiver denial discordance (odds ratio = 0.93, 95% confidence interval = 0.87–0.99).

**Conclusions:**

Discordance between reporters may be crucial for clinical conceptualization. Future research is needed to examine complex longitudinal pathways between family support, ethnic experience and discrimination, and suicidal thoughts discordance among Latinx families.

## 1 | Introduction

Rates of suicidal thoughts and behavior (STB) have been steadily increasing among Hispanic and Latinx populations living in the United States (US) over the past two decades (Curtin et al. 2020; Czeisler et al. 2020), especially among high-school-aged youth (Lindsey et al. 2019; Price and Khubchandani 2022; Xiao et al. 2021). Estimates from a 2023 online survey of 12- to 17-year-old Latinx youth found that 11.8% endorsed past-year suicidal ideation, 8.2% endorsed past-year plan, and 7.7% endorsed past-year attempt (Polanco-Roman et al. 2025). There is need to study STB among US Latinx youth especially given Hispanic and Latinx population growth in the US (United States Census Bureau 2024). Note that while the terms Hispanic and Latinx represent distinct, yet overlapping, populations, the term Latinx will be used unless citing a study that examined populations that the authors referred to as “Hispanic.”

Few grounded suicide theories among racial and ethnic groups have been developed. The Cultural Theory and Model of Suicide for Youth (henceforth labeled “Cultural Theory”), while not specific to Latinx youth, has identified how culture impacts how youth understand suicide, express (or not express) suicide risk, and experience suicide risk in domains of cultural meanings and expressions of stress and suicide, minority stress, and interpersonal conflict (Chu et al. 2022). The Sociocultural Model of Suicide was developed specifically to conceptualize suicide attempts among Latina youth suicide, which focused on the family environment provided through extended family and cultural traditions and beliefs, as well as immediate family functioning (Zayas et al. 2005). Relatedly, a culturally informed suicide risk intervention for Latinx youth emphasize the role of family integration and values (Duarte-Velez et al. 2025; Villarreal-Otálora et al. 2019). Despite the theoretical and clinical emphasis on family and culture, a scoping review identified few studies that included family-level factors or involved family members as predictors of Latinx suicide risk (Villarreal-Otálora et al. 2023), emphasizing the need for more family-centered research.

Gathering data from multiple informants regarding youth suicide risk is common clinical practice. Reports of STB between youth and their caregivers, and the concordance/discordance (or agreement/disagreement in reporting) between them, warrant further investigation to clarify youth suicide risk and inform family-based interventions. As clinician awareness of suicide risk endorsement relies on youth and caregiver report, discordance in reporting complicates clinical intervention. Clinicians need to sensitively assess suicide risk, understand why caregiver-youth reports differ, and adjust treatment accordingly. Identifying factors associated with this discordance may guide research and improve clinical care. Without evidence-based guidance, clinical practice may fail to adequately support suicidal youth and their families.

Research suggests high disagreement between youth and caregivers. Between 37% and 75% of youth endorsed death or suicidal thoughts when parents denied them, and 48%–67% of youth denied when parents endorsed them (Doyle and Fite 2024; Jones et al. 2019; Klaus et al. 2009; Lewis et al. 2014). Predictors of discordance have been examined largely in relation to psychological domains; for example, youth psychopathology has been associated with reduced discordance (Jones et al. 2019; Klaus et al. 2009; LeCloux et al. 2017; Thompson et al. 2006). Of the few studies that have examined ethnicity, between-group rather than within-ethnicity group differences have been explored; these studies suggest greater youth-caregiver discordance among racial and ethnic minority caregivers compared to White caregivers (Jones et al. 2019; Kim et al. 2016; Parker et al. 2025; Thompson et al. 2006). Understanding culturally informed factors associated with discordance in Latinx families is essential to inform responsive assessment and intervention strategies that better support youth with suicide risk and their caregivers.

This study draws on the Cultural Theory and Sociocultural Model of Suicide, which highlight how cultural factors, shaped by family context, impact youth suicide risk and expression. Meaning making of psychological distress, and differences across generations, may affect youth disclosure to caregivers and caregiver awareness of youth risk depending on their own mental health and understanding of youth mental health. Family dynamics, such as cohesion and prioritization (*familismo*), may influence concordance or discordance. Strong family closeness may encourage Latinx youth to share suicidal thoughts (Meca et al. 2021; Vélez-Grau and Lindsey 2022). Similarly, family ethnic socialization (e.g., to *familismo*) and ethnic identity alignment may improve family closeness and reduce discordance (Walker et al. 2022), while family conflict may increase interpersonal stress and hinder youth disclosure of suicide risk. Ethnic discrimination may increase discordance due to greater emotional vulnerability and disconnection from social support (Chu et al. 2022).

Given the cultural relevance of family context to understand youth suicide risk, the current study conducts an exploratory investigation of discordance. We report: (1) discordance between Latinx youth and caregivers related to youth thoughts of death and suicidal thoughts, and (2) the association between demographic, psychological, interpersonal, and ethnicity (i.e., discrimination, identity, and socialization) factors and discordance. For Aim 1, we hypothesize that agreement will be minimal, consistent with past literature outlined above. For Aim 2, we hypothesize youth and parental psychopathology, low family conflict, positive parenting practices, and stronger ethnic identity and ethnic socialization will be associated with reduced discordance. We hypothesize minority stress measured via ethnic discrimination will increase discordance.

## 2 | Methods

### 2.1 | Sample

This study is a secondary analysis of data derived from Project Salud, a Latinx youth-caregiver dyadic study examining protective factors that buffer against ethnic discrimination on youth mental health. Dyads living in one US Midwest city (with approximately 14% Latinx population) were recruited between May and July 2024 from community organizations, schools, churches, and a health network via flyers and email distributions. Inclusion criteria were: (1) youth and caregiver identifying as Latinx/Hispanic, (2) youth ages 13–17 years old, and (3) fluent in either English or Spanish. All study materials were provided in Spanish or English. Consenting dyads completed a self-report survey on REDCap and were compensated with a $50 gift card. All survey data were stored on encrypted devices and secure folders. The study was approved by the Institutional Review Board at Indiana University (Protocol #16925). Data and analytic code will be made available upon reasonable request.

Youth participants who endorsed suicidal ideation were contacted within 24 h for further risk assessment. Caregivers were contacted about their youth’s elevated suicide risk. Resources were provided to the caregivers, and those needing immediate care were encouraged to go to a local emergency department. If contact could not be made, resources were sent to the caregiver via email. Potential suicide risk alerts were generated for 21 youth, but none were assessed at imminent risk and referred to emergency services.

## 3 | Measures

### 3.1 | Demographics

Participants provided information on age and biological sex.

### 3.2 | Youth Psychopathology

#### 3.2.1 | Thoughts of Death and Suicidal Thoughts

The Mood and Feelings Questionnaire Child Self-Report and Parent Report assess depressive symptoms among youth aged 6–17 years old (Angold et al. 1995; Messer et al. 1995; Turner et al. 2014) and has been validated for Spanish-speaking individuals (Gómez-Ortiz et al. 2016). Items indexing thoughts of death and suicidal thoughts included: “In the past 2 weeks,” (1) “[I/She/He] thought that life wasn’t worth living,” (2) “[I/She/He] thought about death and dying,” (3) “[I/She/He] thought my family would be better off without me,” and (4) “[I/She/He] thought about killing myself.” Response options included “Not true” (0), “Sometimes” (1), and “True” (2). All items were dichotomized into no (0) or any (1) endorsement. Of these four items, two items were used in analysis. First, items 1–3 above were combined into no (0) or any (1) thoughts of death or dying. Cronbach’s alpha for combined youth-reported and caregiver-reported thoughts of death were 0.88 and 0.87, respectively. Second, thoughts of killing self (item 4) was retained separately, as thoughts of death is conceptually distinct from suicidal thoughts (Silverman et al. 2007).

#### 3.2.2 | Internalizing and Externalizing Symptoms

The Strengths and Difficulties Questionnaire is a 25-item measure of psychopathology and prosocial behavior used to measure internalizing and externalizing symptoms (Goodman et al. 1998), which has been validated for youth aged 11–16 years old (Goodman et al. 1998) and Spanish-speaking populations (Gaete et al. 2018). Internalizing symptoms were captured with 10 items assessing emotional symptoms (e.g., “I am often unhappy, depressed, or tearful”) and peer problems (e.g., “I have at least one good friend or more”). Externalizing symptoms were captured with 10 items assessing conduct (e.g., “I fight a lot. I can make other people do what I want”) and attention/hyperactivity symptoms (e.g., “I think before I do things”) (Goodman et al. 2010). Response options included “Not true” (0), “Somewhat true” (1), and “Certainly true” (2). Items were reverse coded when appropriate. Mean scores were created separately for internalizing (*α* = 0.76) and externalizing items (*α* = 0.76) with higher scores indicating more symptoms.

#### 3.2.3 | Psychological Treatment

Current treatment was reported by caregivers and asked with the single item, “Is your child currently being treated for any mental health condition (e.g., depression, anxiety, ADHD, autism, ODD, and substance use disorder)?” Caregivers answered “No” (0) or “Yes” (1).

### 3.3 | Caregiver Psychopathology

#### 3.3.1 | Caregiver Anxiety

Generalized Anxiety Disorder-7 is a 7-item measure (e.g., “Feeling nervous”) of generalized anxiety symptoms (Goodman et al. 2010), which has been validated in Spanish-speaking populations (García-Campayo et al. 2010). Caregivers were asked to rate their symptoms over the past 2 weeks with response options on a 4-point Likert scale from “Not at all” (0) to “Nearly every day” (3). The total score (range 0–21) was calculated for caregiver anxiety (*α* = 0.92) with higher scores indicating more anxiety symptoms.

### 3.4 | Interpersonal Factors

#### 3.4.1 | Caregiver-Child Conflict

Family conflict was measured with a 16-item, youth self-report questionnaire of disagreements between youth and caregivers in various domains, such as “chores (like helping around the house),” “friends (like choice of friends, what you do with friends),” and “cultural traditions (like not wanting to follow your parents’ traditions)” (Padilla et al. 2016; Smetana et al. 2009; Updegraff et al. 2012). Youth were asked to consider the past year with response options on a 6-point Likert scale from “Not at all” (1) to “Several times a day” (6). The mean score was calculated (*α* = 0.87) with greater scores indicating more caregiver-youth conflict.

#### 3.4.2 | Parenting Practices

Caregiver parenting behaviors reported by youth were measured across two subscales from the Parental Practices and Communication Scale (Gorman-Smith et al. 1996), which has been validated for Latinx youth (Orpinas et al. 1999). The monitoring and involvement subscale measures caregiver involvement across 11 items, such as “In the past 30 days, how often did a parent have time to listen to you when you wanted to talk with one of them.” Youth rated items on a 5-point Likert scale from “Don’t know or never” (1) to “Yesterday/today” (5). The positive parenting subscale measures the use of positive rewards and encouragement for appropriate behavior across 6 items, such as “How often did one of them say something nice about you?” Items were also on a 5-point Likert scale from “Almost never” (1) to “Almost always” (5). Mean scores for monitoring and involvement (*α* = 0.88) and for positive parenting (*α* = 0.91) were calculated with higher scores indicating more monitoring/involvement and positive parenting, respectfully.

#### 3.4.3 | Family Support

The Multidimensional Scale of Perceived Social Support is a 12-item measure of perceived social support from family (Zimet et al. 1988), which has been validated among Spanish-speaking youth in Colombia (Trejos-Herrera et al. 2018). We used the family (4 items, “My family really tries to help me”) subscale. Youth were asked to rate the extent to which they agreed or disagreed on a 7-point Likert scale from “Very strongly disagree” (1) to “Very strongly agree” (7). The mean score was calculated (*α* = 0.95) with higher scores indicating more perceived family social support.

### 3.5 | Ethnic Discrimination, Identity, and Socialization

#### 3.5.1 | Ethnic Discrimination and Coping

The Everyday Discrimination scale is a 9-item measure that assessed the frequency of day-to-day experiences of discrimination (Williams et al. 1997). Youth were asked how often discrimination experiences occur “because of your race or ethnicity.” Items (e.g., “You are treated with less courtesy that other people are”) were rated on a 6-point Likert Scale from “Never” (0) to “Almost every day” (5). A total score was calculated for experiences of discrimination (*α* = 0.93) where higher scores represented more frequent experiences of ethnic discrimination.

The proactive coping subscale of the Discrimination Coping Scale is a 3-item measure that assessed youth’s ability to engage in strategies and behaviors to prevent or prepare for potential experiences of discrimination (e.g., “How often have you used the strategy of talking to the person to clarify the misconception or stereotype?”) (Umaña-Taylor et al. 2008). Youth rated items on a 5-item Likert scale from “Never” (1) to “Very often” (5). A total score was calculated for proactive coping (*α* = 0.71), with higher scores representing more time spent preparing for experiences of discrimination.

#### 3.5.2 | Ethnic Identity

The Ethnic Identity Scale is a 17-item measure of attitudes toward one’s ethnicity, defined as “cultural traditions, beliefs, and behaviors that are passed down through generations” (Umaña-Taylor et al. 2004). Youth were asked to respond to items on three subscales: (1) exploration (“I have experienced things that reflect my ethnicity, such as eating food, listening to music, and watching movies”), (2) resolution (“I know what my ethnicity means to me”), and (3) affirmation (“My feelings about my ethnicity are mostly negative”). Response options included a 4-point Likert from “Does not describe me at all” (1) and “Describes me very well” (4). Items were reversed scored when appropriate. Mean scores were calculated for each subscale, with higher scores indicating greater exploration (*α* = 0.86), resolution (*α* = 0.91), and affirmation (*α* = 0.80) of one’s ethnic identity.

#### 3.5.3 | Ethnic Socialization

The Familial Ethnic Socialization Measure is a 12-item measure of the extent to which respondents perceive their families to have socialized them to their ethnic identities and was originally developed in a sample of adolescents of Mexican origin (Umaña-Taylor and Fine 2001). Youth were asked to rate items (e.g., “My family participates in activities that are specific to my ethnic/cultural background”) on a 5-point Likert from “Not at all” (1) to “Very much” (5). The mean score was calculated for ethnic socialization (*α* = 0.93) with higher scores indicating greater perceived ethnic socialization.

### 3.6 | Analyses

We conducted descriptive cross-tabulations between any thoughts of death/dying and suicidal thoughts and caregiver and/or youth endorsement of items. Additionally, we calculated percent agreement and Cohen’s Kappa to measure level of agreement between youth and caregivers (Aim 1). Cohen’s Kappa accounts for chance agreement between raters and ranges from −1 to +1 (McHugh 2012). Guidelines for interpreting *κ* state that scores below 0.20 have no agreement, 0.20–0.39 are minimal, 0.40–0.59 are weak, 0.60–0.79 are moderate, 0.80–0.90 are strong, and greater than 0.90 are nearly perfect (McHugh 2012). Cohen’s kappa is often recommended as a more reliable estimate compared to percent agreement, especially when percent agreement may be inflated by the high prevalence of an observed outcome—in this study, an expected high prevalence of concordance between youth-caregivers (i.e., zeros) (Bryington et al. 2002).

Univariable logistic regression models were calculated between psychosocial measures and discordance (Aim 2). Discordance was defined in two ways: (a) youth endorsement and caregiver denial (labeled caregiver denial), and (b) youth denial and caregiver endorsement (labeled youth denial). Univariable analyses were conducted as the second aim was descriptive, as prior research has not examined study variables in relation to Latinx youth-caregiver discordance. Sensitivity analyses analyzed the thoughts of death items individually for Aims 1 and 2.

## 4 | Results

### 4.1 | Participant Characteristics

Among the 127 dyads who participated in the larger study, 7 (5.5%) were removed for incomplete data on items assessing thoughts of death or suicidal thoughts. The final analytic sample included 120 child-caregiver dyads. Youth on average were 14.99 years old (standard deviation = 1.52) and were majority female biological sex (61.7%). Most Latinx youth identified their nationality as either Mexican (55.3%) or American (25.0%). Maternal caregivers completed most surveys (88.3%), followed by paternal caregivers (8.4%). The remaining caregivers did not indicate their relation to their youth (3.3%).

Table 1 provides a summary of youth and caregiver endorsed thoughts of death and suicidal thoughts. Nearly a quarter of youth endorsed having thoughts of death or dying (24.2%). Fewer youth endorsed having thoughts of killing self (13.3%). Caregivers endorsed items less frequently: any thoughts of death (12.5%) and thoughts of killing self (7.6%).

### 4.2 | Aim 1: Discordance Between Youth and Caregivers

Table 1 also depicts the prevalence of caregiver and youth discordance in their reports of thoughts of death and suicidal thoughts. Most caregivers and youth reported concordant-denial (i.e., agreement in denial) thoughts of death (70.0%), while 23.3% of caregiver-youth dyads had discordant reporting on thoughts of death (caregiver denial: 17.5%; child denial: 5.8%). Few had concordant-endorsement (i.e., agreement in endorsement) thoughts of death (6.7%). Kappa estimates revealed minimal agreement between youth and caregivers for thoughts of death (*κ* = 0.24, *p* = 0.005).

Most caregivers and youth also reported concordant-negative thoughts of killing self (84.3%), while 10.8% of caregiver-youth dyads had discordant reporting on thoughts of killing self (caregiver denial: 8.3%; child denial: 2.5%). Few had concordant-positive thoughts of killing self (5.0%). Kappa estimates revealed weak agreement for thoughts of killing self (*κ* = 0.43, *p* < 0.01).

### 4.3 | Aim 2: Factors Associated With Discordance

#### 4.3.1 | Caregiver Denial

Table 2 summarizes the results from univariable logistic regression analyses predicting caregiver denial. Results revealed that child psychopathology, interpersonal factors, and ethnic discrimination and socialization were associated with caregiver denial discordance.

##### 4.3.1.1 | Psychopathology

Child internalizing was associated with an increased odds of caregiver denial of thoughts of death or dying (OR = 1.33, 95% CI: 1.15, 1.53) and thoughts of killing self (OR = 1.28, 95% CI: 1.08, 1.53). Child externalizing was also associated with increased odds of caregiver denial of thoughts of death or dying (OR = 1.39, 95% CI: 1.17, 1.65) and thoughts of killing self (OR = 1.43, 95% CI: 1.12, 1.82).

##### 4.3.1.2 | Interpersonal Factors

Family conflict was positively associated with an increased odds of caregiver denial of thoughts of death or dying (OR = 4.48, 95% CI: 2.00, 10.07) and thoughts of killing self (OR = 6.67, 95% CI: 1.91, 23.31). Family support, parental monitoring, and positive parenting were protective against both thoughts of death or dying (OR_range_ = 0.28–0.53) and thoughts of killing self (OR_range_ = 0.19–0.51).

##### 4.3.1.3 | Ethnic Discrimination, Identity, and Socialization

Ethnic discrimination was associated with an increased odds of caregiver denial of thoughts of death or dying (OR = 1.06, 95% CI: 1.01, 1.11) and thoughts of killing self (OR = 1.06, 95% CI: 1.00, 1.13). Ethnic socialization was only protective against discordance for thoughts of killing self (OR = 0.93, 95% CI = 0.87–0.99).

#### 4.3.2 | Youth Denial

Table 3 summarizes the results from univariable logistic regression analyses predicting youth denial.

##### 4.3.2.1 | Psychopathology

Both child internalizing (OR = 1.28, 95% CI: 1.08, 1.53) and externalizing psychopathology (OR = 1.43, 95% CI: 1.12, 1.82) were associated with thoughts of killing self, but not thoughts of death or dying.

##### 4.3.2.2 | Interpersonal Factors

Family support was associated with lower odds of child denial of thoughts of death or dying (OR = 0.60, 95% CI: 0.39, 0.92) and thoughts of killing self (OR = 0.51, 95% CI: 0.34, 0.76). Both parental monitoring (OR = 0.19, 95% CI: 0.07, 0.52) and positive parenting (OR = 0.47, 95% CI: 0.25, 0.88) were associated with lower odds of child denial of thoughts of killing self. Family conflict was associated with higher odds of child denial of thoughts of killing self (OR = 6.67, 95% CI: 1.91, 23.31).

##### 4.3.2.3 | Ethnicity Discrimination, Identity, and Socialization

Ethnic socialization was associated with lower odds of child denial of thoughts of death or dying (OR = 0.36, 95% CI: 0.17, 0.78) and thoughts of killing self (OR = 0.93, 95% CI: 0.87, 0.99). Discrimination was associated with higher odds of child denial of thoughts of killing self (OR = 1.06, 95% CI: 1.00, 1.13). For all above analyses, Appendices 1–3 present individual-item analyses.

## 5 | Discussion

The current study aimed to describe the extent of youth-caregiver thoughts of death and suicidal thoughts discordance, as well as examine associations between demographic, psychopathology, social support, parenting, ethnic discrimination, ethnic identity, and ethnic socialization with discordance.

### 5.1 | Aim 1: Discordance Between Youth and Caregivers

Estimates between caregivers and youth were highest for thoughts of killing self and lowest for thoughts that life is not worth living. Higher kappa values for thoughts of killing self relative to thoughts of death/dying are consistent with a prior study among youth aged 11–17 recruited from primary care (Jones et al. 2019). Notably, this prior study excluded individuals who were not proficient in English, further attesting to the need to extend work on Latinx youth-caregiver discordance. We suppose that kappa estimates were higher for thoughts of killing self as fewer individuals endorsed this item and, thus, agreement in which both parties in the dyad did not endorse the item was more prevalent. However, despite the differences across the thoughts of death and suicidal thoughts items, kappa estimates generally demonstrated minimal to weak agreement.

The minimal agreement between youth and caregivers on thoughts of death and suicidal thoughts in this study is generally comparable with past research (Brahmbhatt and Grupp-Phelan 2019). For example, only 40% of caregivers identified their suicidal youth as suicidal in past research (Lewis et al. 2014). Although findings from our study suggest that youth endorse suicidal thoughts at higher prevalences compared to caregiver report, a small group of caregivers endorsed items when youth denied. Information regarding suicide risk (or global mental health functioning) may be missed when conducting assessment with only one informant.

Examining ethnicity as a predictor of discordance may yield contradictory results. For example, Jones et al. (2019) found that Latinx youth were less likely than non-Latinx youth to experience parental denial discordance on thoughts of death, but not suicidal thoughts (Jones et al. 2019). While our study did not examine non-Latinx youth-caregiver dyads for direct comparison, our results indicated higher agreement on suicidal ideation than on thoughts of death. Further research with Latinx dyads should replicate this finding to clarify discordance on youths’ thoughts through a cultural lens. For example, it is possible that thoughts of death may be more culturally acceptable among Latinx dyads living in the Midwest US, thus leading to greater disclosure and concordance. It is also possible that thoughts of suicide may be more severe (potentially indicated through greater mental health intervention), which inevitably results in greater caregiver awareness. The degree of closeness and trust with caregivers, with moderators of ethnic socialization, in relation to discordance is an important future research direction to elucidate cultural differences between thoughts of death and suicidal thoughts.

### 5.2 | Aim 2: Factors Associated With Discordance

#### 5.2.1 | Demographics

With respect to our second aim, we investigated demographic, youth and caregiver psychopathology, interpersonal, and ethnicity factors and found that variables in all but the demographic domain were associated with discordance. However, when examining thoughts of death items separately in a sensitivity analysis (Appendices 2 and 3), the only demographic factor that demonstrated associations with discordance was age. Differences between main and sensitivity analyses (i.e., combined vs. individual thoughts of death items) may be due to Type I errors with increased number of comparisons or reduced variance in the combined variable. However, age, and the corresponding broader developmental period, may be relevant to the context of discordance. Consistent with this, a prior study of youth aged 11–17 found a negative association between age and caregiver denial-youth endorsement on thoughts of death and suicidal thoughts (Jones et al. 2019)—a trend replicated in the current study.

The Sociocultural Model of Suicide identifies the importance of adolescent development to suicide risk. Adolescents’ desire for increased autonomy may be at odds with disclosure to caregivers (Chu et al. 2022; Zayas et al. 2005); however, our results do not support this hypothesis. Rather, as youth age, youth may be more capable or willing to disclose thoughts. Interestingly, age was positively associated with youth denial-caregiver endorsement discordance of life not worth living. If youth do not disclose, caregivers may know that their youth is struggling but may not know youths’ specific thoughts. Maintaining skepticism regarding our null findings in the main results, future research would benefit from examining changes in discordance by year-age among Latinx youth and its association with other variables (e.g., cultural beliefs, family cohesion, and ethnic identity).

#### 5.2.2 | Youth and Caregiver Psychopathology

Regarding psychopathology factors, our results are contradictory to our hypotheses and past research (Jones et al. 2019; Klaus et al. 2009). For youth endorsement-caregiver denial discordance, past research found reduced odds with family history of suicide, psychoactive medications, psychotherapy, and prior hospitalizations. For youth denial-caregiver endorsement discordance, general psychopathology, symptom severity, and prior hospitalization were associated with reduced odds (Jones et al. 2019). Our study demonstrated the reverse—youth internalizing and externalizing symptoms were associated with increased odds of discordance, specifically between youth endorsement and caregiver denial. We conducted univariable analyses, whereas Jones et al. (2019) conducted multivariable analyses with clinical factors. It is possible that our observed association may be attributed to confounding factors.

Alternatively, the discrepancy between past research and the current findings may reflect cultural differences, such as suicide stigma between Latinx dyads in a community sample and non-Latinx families. As suggested by prior research among Hispanic individuals, stigma toward suicide and help-seeking may be more common than among non-Hispanic populations (Oquendo et al. 2005; Silva and Van Orden 2018). Greater youth-reported internalizing and externalizing symptoms may be related to greater caregiver-youth discordance through stigmatizing beliefs toward mental health, thoughts of death, and suicidal thoughts.

Research also suggests that caregiver ethnic identity and acculturation, specifically the assimilation into the host culture, may play an important role in beliefs toward mental health. Prior research among US Latinx caregivers found that youth with internalizing problems had greater probability of service use when caregivers endorsed greater acculturation (Galvan and Gudiño 2021). There is likely a complex association between youths’ thoughts of death and suicide, disclosure of suicidal thoughts to caregivers, and ethnic identity and acculturation that warrants further investigation. This interpretation is in line with the Cultural Theory, suggesting that cultural meaning and expression impact suicide risk (Chu et al. 2022). Therefore, cultural values (both within family and within host culture) regarding mental health and suicide may be affecting discordance among the sample.

#### 5.2.3 | Interpersonal Factors

In line with study hypotheses, results indicated that greater family conflict was associated with increased odds of discordance, while family support, parental monitoring, and positive parenting strategies were associated with reduced odds of discordance. These findings replicate prior research and are consistent within the Cultural Theory and Sociocultural Theory of Suicide (Chu et al. 2022; Zayas et al. 2005), which both emphasize family cohesion and strong caregiver relationships as protective factors against youth suicide risk. For example, Klaus et al. (2009) found a negative association between family support and youth endorsement-caregiver denial discordance of youth suicide plan and suicide attempt among a clinical adolescent sample (Klaus et al. 2009). Similarly, Kim et al. (2016) found that parenting practices that prioritized connection and youth autonomy are associated to lower levels of discordance in depression – which often co-occurs with suicidality – among racial and ethnic minority youth (Kim et al. 2016). Together, these findings suggest that supportive, autonomy-promoting family environments may foster greater caregiver awareness and acknowledgment of youths’ suicide-related risks.

#### 5.2.4 | Ethnic Discrimination, Identity, and Socialization

Ethnic discrimination was associated with higher odds of discordance in line with hypotheses. Generally, experiences of discrimination are associated with increased suicidal thoughts among Latinx populations (Kwon and Han 2019), therefore, the likelihood of caregiver-youth discordance may also increase. Chronic exposure to discrimination may exacerbate youths’ psychological distress while simultaneously complicating the disclosure of suicidal thoughts, as youth may fear negative or invalidating reactions from caregivers (Galán et al. 2021; Shin et al. 2025). As a result, adolescents may be more likely to internalize distress rather than seek support, increasing the risk that caregivers remain unaware of their youth’s suicidal thoughts.

Ethnic identity development is another central developmental process during adolescence with important implications for youth well-being (Phinney 1989), and is closely interconnected with family ethnic socialization (Constante et al. 2020). Results revealed that ethnic identity and ethnic socialization were associated with lower odds of discordance consistent with study hypotheses. The Cultural Theory proposes that stress related to ethnic identity is predictive of risk (Chu et al. 2022). Low ethnic identity among Latinx young adults has been associated with suicide risk through feelings of burdensomeness (Oakey-Frost et al. 2021); this may extend to discordance if youth view disclosure as a burden to caregivers. The Sociocultural Theory of Suicide posits that strong *familismo* values may enhance family closeness (Zayas et al. 2005), which then may reduce discordance. Empirical work demonstrates that ethnic socialization is associated with improved mental health outcomes (Ayón et al. 2020). Alternatively, withdrawal from culturally relevant and valued activities is associated with a lower sense of belongingness and greater suicidal thoughts (Silva and Van Orden 2018). Among Latinx youth, strong identification with *familismo* reduced the impact of depression on suicidal thoughts (Walker et al. 2022). Ethnic identity and socialization may offer points of clinical intervention to strengthen connection (and reduce discordance) between youth and caregivers.

### 5.3 | Clinical Implications

From a clinical perspective, our findings further emphasize the need for multiple informants on youth suicide risk. The familial context of discordance is crucial in conceptualization of a youth’s suicide risk and treatment plan. Non-reductive exploration of youth, family, and cultural beliefs about suicide and mental health will facilitate a nuanced tailoring of intervention strategies to specifically address youth suicide risk (Duarté-Vélez et al. 2024; Walker et al. 2022; Williams et al. 1997). It is important to note that very little research has been conducted on developing and studying interventions to reduce suicide risk among Latinx youth and the cultural responsiveness of interventions that do exist is minimal (Villarreal-Otálora et al. 2019). Discordance between youth and caregivers may be a relevant domain to integrate into future intervention development. Arguably, however, increasing thorough suicide assessment from youth and caregivers in non-suicide-focused treatment is needed as seeking treatment for STB may reduce discordance.

## 6 | Strengths and Limitations

The results are contextualized within minimal suicide research among Latinx populations, which has been highlighted by past researchers as potentially due to the “Latinx Paradox,” in which the lack of higher suicide rates among Latinx populations in the face of other health disparities reduces the public health imperative to study suicide among Latinx populations (Silva and Van Orden 2018). Research is needed to extend beyond looking at ethnicity as a predictor of discordance and examine the experiences within ethnic groups associated with discordance. Our study meaningfully adds to the literature through its analysis of numerous domains that are theoretically relevant to Latinx youth suicide risk and discordance with caregiver report. Our research supports the direction of future discordance research to examine interpersonal factors and ethnic identity, discrimination, and socialization. Cultural domains that enhance ethnic identity and cultural socialization may be protective against discordance; research is needed to examine how ethnic identity and socialization domains can address discordance in clinical settings. We also highlight the strength and importance of data collection among a community sample of bilingual or monolingual Spanish speakers.

Results of this study must be understood considering its limitations. We prioritized the use of validated measures among Spanish-speaking populations that did not include details on the intensity and duration of suicidal thoughts. Future studies may benefit from examining discordance using more detailed suicide measures (e.g., Beck Scale for Suicide Ideation). Given the small sample size, we were underpowered to examine differences between Latinx ethnic subgroups. Although combining groups conserves power, it also assumes equivalent risk across ethnicities. This is noteworthy given prior evidence of meaningful differences in STB among those identifying with Puerto Rican ethnicity compared to Mexican and Cuban identities (Oquendo et al. 2004; Ungemack and Guarnaccia 1998). Larger, subgroup-specific samples are needed to determine potential differences in caregiver-youth discordance. Consistent with prior research (Jones et al. 2019; Kim et al. 2016), most caregivers in the sample were mothers, which may limit generalizability to other caregiver types (e.g., fathers or non-parental caregivers). Analyses were also limited to univariable regression due to the exploratory nature of the investigation; future studies should examine multivariable predictors of discordance. Finally, we included a broad measure of ethnic socialization (e.g., awareness, participation, and pride in ethnic background), but did not capture specific cultural values (e.g., *familismo*) which are likely multidimensional constructs warranting greater investigation in relation to discordance (Walker et al. 2022). We also did not include a measure of acculturation, which may be particularly informative to understand discordance dependent on identification with host culture and beliefs around youth suicide.

## 7 | Conclusions

Given the large gap in our understanding of thoughts of death/suicidal thoughts discordance between Latinx youth and caregivers, the current study aimed to describe the level of discordance, as well as factors associated with discordance. We found minimal agreement between youth and caregivers, as well as associations between youth internalizing and externalizing symptoms, family support, parenting factors, ethnic discrimination, and ethnic socialization with discordance. Future research is needed to examine these relations within a longitudinal context.

## Supplementary Material

Supplementary Materials_Appendix 1 through 3

Additional supporting information can be found online in the Supporting Information section.

SuicidalityConcordance_Supplement_JoA_20260226.

## Figures and Tables

**TABLE 1 |:** Endorsement of thoughts of death and suicidal thoughts.

	Youth endorsement (*N*, %)	Caregiver endorsement (*N*, %)	Youth endorsement; Caregiver denial (*N*, %)	Youth denial; Caregiver endorsement (*N*, %)	Overall kappa

Any thoughts of death/dying	29 (24.2)	15 (12.5)	21 (17.5)	7 (5.8)	0.24*
Thoughts of killing self	16 (13.3)	9 (7.6)	10 (8.3)	3 (2.5)	0.43**

*Note:* Data from 120 youth‐caregiver dyads.

* *p* < 0.05;

** *p* < 0.001.

**TABLE 2 |:** Univariable logistic regression analyses associated with caregiver denial.

	Thoughts of death/dying^a^		Thoughts of killing self^b^	
	*n*	OR (95% CI)	*n*	OR (95% CI)

Demographics				
Age	113	0.59 (0.65, 1.28)	115	1.10 (0.70, 1.73)
Female biological sex	113	1.74 (0.58, 5.21)	114	2.07 (0.41, 10.46)
Psychopathology				
Child internalizing	113	**1.33 (1.15, 1.53)**	113	**1.28 (1.08, 1.53)**
Child externalizing	113	**1.39 (1.17, 1.65)**	112	**1.43 (1.12, 1.82)**
Child in treatment	113	0.54 (0.11, 2.57)	117	0.59 (0.07, 4.95)
Caregiver anxiety	113	1.07 (0.98, 1.17)	108	1.07 (0.95, 1.21)
Interpersonal				
Family conflict	113	**4.48 (2.00, 10.07)**	109	**6.67 (1.91, 23.31)**
Family support	113	**0.53 (0.37, 0.76)**	114	**0.51 (0.34, 0.76)**
Parental monitoring	113	**0.28 (0.14, 0.56)**	113	**0.19 (0.07, 0.52)**
Positive parenting	113	**0.47 (0.30, 0.76)**	111	**0.47 (0.25, 0.88)**
Ethnic discrimination, identity, and socialization				
Discrimination	113	**1.06 (1.01, 1.11)**	113	**1.06 (1.00, 1.13)**
Proactive coping	113	0.92 (0.79, 1.08)	115	0.92 (0.75, 1.15)
Ethnic identity exploration	113	0.75 (0.40, 1.40)	104	0.74 (0.32, 1.71)
Ethnic identity affirmation	113	1.85 (0.63, 5.45)	109	0.98 (0.33, 2.88)
Ethnic identity resolution	113	0.72 (0.41, 1.29)	110	0.84 (0.38, 1.89)
Racial/ethnic socialization	113	0.68 (0.39, 1.18)	112	**0.93 (0.87, 0.99)**

*Note:* Sample sizes within columns vary due to incomplete data in predictor variable. Bolded estimates represent significant at *p* < 0.05.

a Based on 113 dyads, including 92 caregiver‐youth dyads in agreement and 21 discordant dyads where youth endorse SI and caregiver denies.

b Based on 117 dyads, including 107 caregiver‐youth dyads in agreement and 10 discordant dyads where youth endorse SI and caregiver denies.

**TABLE 3 |:** Univariable logistic regression analyses associated with child denial.

	Thoughts of death/dying^a^		Thoughts of killing self^b^	
	*n*	OR (95% CI)	*n*	OR (95% CI)

Demographics				
Age	99	1.57 (0.88, 2.78)	115	1.10 (0.70, 1.73)
Female biological sex	99	0.29 (0.50, 1.17)	114	2.07 (0.41, 10.46)
Psychopathology				
Child internalizing	99	1.13 (0.92, 1.39)	113	**1.28 (1.08, 1.53)**
Child externalizing	99	1.15 (0.92, 1.43)	112	**1.43 (1.12, 1.82)**
Child in treatment	99	2.05 (0.36, 11.59)	117	0.59 (0.07, 4.95)
Caregiver anxiety	99	1.07 (0.93, 1.23)	108	1.07 (0.95, 1.21)
Interpersonal				
Family conflict	99	2.05 (0.77, 5.45)	109	**6.67 (1.91, 23.31)**
Family support	99	**0.60 (0.39, 0.92)**	114	**0.51 (0.34, 0.76)**
Parental monitoring	99	0.54 (0.23, 1.28)	113	**0.19 (0.07, 0.52)**
Positive parenting	99	0.56 (0.26, 1.19)	111	**0.47 (0.25, 0.88)**
Ethnic discrimination, identity, and socialization				
Discrimination	99	1.05 (0.98, 1.13)	113	**1.06 (1.00, 1.13)**
Proactive coping	99	0.94 (0.74, 1.20)	115	0.92 (0.75, 1.15)
Ethnic identity exploration	99	0.52 (0.18, 1.49)	104	0.74 (0.32, 1.71)
Ethnic identity affirmation	99	0.64 (0.23, 1.78)	109	0.98 (0.33, 2.88)
Ethnic identity resolution	99	0.49 (0.19, 1.25)	110	0.84 (0.38, 1.89)
Racial/ethnic socialization	99	**0.36 (0.17, 0.78)**	112	**0.93 (0.87, 0.99)**

*Note:* Sample sizes within columns vary due to incomplete data in predictor variable. Bolded estimates represent significant at *p* < 0.05.

a Based on 99 dyads, including 92 caregiver‐youth dyads in agreement and 7 discordant dyads where youth endorse SI and caregiver denies.

b Based on 117 dyads, including 107 caregiver‐youth dyads in agreement and 10 discordant dyads where youth endorse SI and caregiver denies.

## Data Availability

The data that support the findings of this study are available from the corresponding author upon reasonable request.
