# Supplementary material for: Discordance Between Thoughts of Death and Suicidal Ideation Among Latinx Youth and Caregivers in the United States
Source: J Adolesc. Author manuscript; Available in PMC 2026 May 22. (PMC13191574; doi:10.1002/jad.70143)
Supplement: Supplementary Materials_Appendix 1 through 3 [file NIHMS2168364-supplement-Supplementary_Materials_Appendix_1_through_3.docx]

**Appendix**

**Appendix 1.** Endorsement of thoughts of death and suicidal thoughts using individual items.

**Appendix 2.** Univariable logistic regression analyses associated with caregiver denial using individual items.

**Appendix 3.** Univariable logistic regression analyses associated with youth denial using individual items.

| **Appendix 1. Endorsement of thoughts of death and suicidal thoughts using individual items.** | | | | | |
| --- | --- | --- | --- | --- | --- |
|  | Youth Endorsement (N, %) | Caregiver Endorsement  (N, %) | Youth Endorsement; Caregiver Denial (N, %) | Youth Denial; Caregiver Endorsement (N, %) | Overall Kappa |
| Life not worth living^a^ | 23 (19.5) | 14 (11.9) | 17 (14.4) | 8 (6.8) | .21* |
| Thoughts of death/dying^a^ | 20 (16.9) | 8 (6.8) | 15 (12.7) | 3 (2.5) | .29** |
| Family being better off^a^ | 21 (17.8) | 7 (5.9) | 18 (15.3) | 4 (3.4) | .14 |
| Thoughts of killing self^b^ | 16 (13.6) | 9 (7.6) | 10 (8.3) | 3 (2.5) | .43** |
| *Notes.* ^a^data from 118 youth-caregiver dyads; ^b^data from 120 youth-caregiver dyads; *p<.05, **p<.001 | | | | | |

| **Appendix 2. Univariable logistic regression analyses associated with caregiver denial using individual items.** | | | | | | | | |
| --- | --- | --- | --- | --- | --- | --- | --- | --- |
|  | Life not worth living^a^ | | Thoughts of death/dying^b^ | | Family being better off^c^ | | Thoughts of killing self^d^ | |
|  | n | OR (95% CI) | n | OR (95% CI) | n | OR (95% CI) | n | OR (95% CI) |
| Demographics |  |  |  |  |  |  |  |  |
| Age | 108 | 0.91 (0.62, 1.32) | 113 | **0.61 (0.39, 0.95)** | 112 | 0.89 (0.64, 1.26) | 115 | 1.10 (0.70, 1.73) |
| Female biological sex | 106 | 2.51 (0.66, 9.45) | 113 | 3.80 (0.81, 17.92) | 110 | 2.25 (0.69, 7.38) | 114 | 2.07 (0.41, 10.46) |
| Psychopathology |  |  |  |  |  |  |  |  |
| Child internalizing | 106 | **1.32 (1.14, 1.54)** | 111 | **1.37 (1.16, 1.61)** | 110 | **1.34 (1.45, 1.57)** | 113 | **1.28 (1.08, 1.53)** |
| Child externalizing | 106 | **1.39 (1.15, 1.67)** | 110 | **1.44 (1.17, 1.78)** | 109 | **1.55 (1.26, 1.92)** | 112 | **1.43 (1.12, 1.82)** |
| Child in treatment | 110 | 0.33 (0.04, 2.64) | 115 | 0.87 (0.18, 4.26) | 114 | 1.28 (0.32, 5.03) | 117 | 0.59 (0.07, 4.95) |
| Caregiver anxiety | 101 | 1.09 (0.99, 1.20) | 106 | 1.09 (0.99, 1.21) | 105 | 1.04 (0.95, 1.15) | 108 | 1.07 (0.95, 1.21) |
| Interpersonal |  |  |  |  |  |  |  |  |
| Family conflict | 103 | **5.59 (2.16, 14.43)** | 107 | **3.38 (1.47, 7.78)** | 106 | **3.24 (1.55, 6.79)** | 109 | **6.67 (1.91, 23.31)** |
| Family support | 107 | **0.53 (0.36, 0.78)** | 112 | **0.65 (0.46, 0.91)** | 111 | **0.58 (0.41, 0.81)** | 114 | **0.51 (0.34, 0.76)** |
| Parental monitoring | 106 | **0.27 (0.13, 0.58)** | 111 | **0.24 (0.10, 0.54)** | 110 | **0.30 (0.15, 0.61)** | 113 | **0.19 (0.07, 0.52)** |
| Positive parenting | 106 | **0.53 (0.33, 0.87)** | 108 | **0.60 (0.36, 0.99)** | 107 | **0.51 (0.31, 0.84)** | 111 | **0.47 (0.25, 0.88)** |
| Ethnicity Related Factors |  |  |  |  |  |  |  |  |
| Discrimination | 106 | **1.06 (1.01, 1.12)** | 111 | **1.05 (1.00, 1.11)** | 110 | **1.05 (1.01, 1.11)** | 113 | **1.06 (1.00, 1.13)** |
| Proactive coping | 109 | 0.91 (0.76, 1.08) | 113 | 1.02 (0.85, 1.22) | 112 | 0.99 (0.84, 1.16) | 115 | 0.92 (0.75, 1.15) |
| Ethnic identity  exploration | 98 | 0.59 (0.31, 1.14) | 101 | 0.83 (0.41, 1.68) | 84 | 0.84 (0.43, 1.66) | 104 | 0.74 (0.32, 1.71) |
| Ethnic identity  affirmation | 103 | 1.57 (0.51, 4.80) | 108 | 0.79 (0.34, 1.82) | 107 | 1.79 (0.56, 5.67) | 109 | 0.98 (0.33, 2.88) |
| Ethnic identity  resolution | 105 | 0.68 (0.37, 1.26) | 108 | 0.67 (0.34, 1.34) | 107 | 0.70 (0.36, 1.36) | 110 | 0.84 (0.38, 1.89) |
| Racial/ethnic  socialization | 105 | 0.95 (0.90, 1.00) | 110 | 0.94 (0.89, 1.00) | 109 | 0.96 (0.92, 1.01) | 112 | **0.93 (0.87, 0.99)** |
| *Notes.* Sample sizes within columns vary due to incomplete data in predictor variable. Bolded estimates represent significant at p<0.05.  ^a^Based on 110 dyads including 93 caregiver-youth dyads in agreement and 17 discordant dyads where youth endorse SI and caregiver denies.  ^b^Based on 115 dyads including 100 caregiver-youth dyads in agreement and 15 discordant dyads where youth endorse SI and caregiver denies.  ^c^Based on 114 dyads including 96 caregiver-youth dyads in agreement and 18 discordant dyads where youth endorse SI and caregiver denies.  ^d^Based on 117 dyads including 107 caregiver-youth dyads in agreement and 10 discordant dyads where youth endorse SI and caregiver denies. | | | | | | | | |

| **Appendix 3. Univariable logistic regression analyses associated with youth denial using individual items.** | | | | | | | | |
| --- | --- | --- | --- | --- | --- | --- | --- | --- |
|  | Life not worth living^a^ | | Thoughts of death/dying^b^ | | Family being better off^c^ | | Thoughts of killing self^d^ | |
|  | n | OR (95% CI) | n | OR (95% CI) | n | OR (95% CI) | n | OR (95% CI) |
| Demographics |  |  |  |  |  |  |  |  |
| Age | 100 | **2.27 (1.17, 4.39)** | 102 | 1.14 (0.52, 2.50) | 98 | 1.64 (0.77, 3.46) | 109 | 1.40 (0.64, 3.10) |
| Female biological sex | 98 | 0.58 (0.14, 2.47) | 100 | Not Stable | 96 | 1.93 (0.19, 19.27) | 107 | 0.59 (0.04, 9.72) |
| Psychopathology |  |  |  |  |  |  |  |  |
| Child internalizing | 97 | 1.20 (0.97, 1.48) | 99 | 1.16 (0.87, 1.56) | 96 | 1.25 (0.97, 1.62) | 106 | 1.05 (0.77, 1.43) |
| Child externalizing | 97 | 1.23 (0.98, 1.55) | 99 | 1.23 (0.88, 1.71) | 96 | 1.28 (0.94, 1.73) | 106 | 1.03 (0.75, 1.42) |
| Child in treatment | 101 | 3.12 (0.67, 14.47) | 103 | 11.33 (0.97, 132.98) | 100 | **19.15 (1.85, 198.34)** | 110 | 2.64 (0.23, 30.85) |
| Caregiver anxiety | 93 | 1.05 (0.91, 1.21) | 95 | 1.07 (0.88, 1.30) | 93 | 1.01 (0.84, 1.23) | 102 | 1.05 (0.86, 1.29) |
| Interpersonal |  |  |  |  |  |  |  |  |
| Family conflict | 96 | 2.24 (0.87, 5.76) | 97 | 1.49 (0.38, 5.92) | 93 | 1.19 (0.37, 3.83) | 103 | 1.04 (0.27, 4.08) |
| Family support | 101 | **0.58 (0.38, 0.89)** | 102 | 0.96 (0.44, 2.09) | 98 | **0.45 (0.26, 0.81)** | 108 | 1.38 (0.50, 3.87) |
| Parental monitoring | 98 | 0.48 (0.21, 1.09) | 101 | 0.37 (0.11, 1.24) | 97 | **0.27 (0.09, 0.85)** | 107 | 0.80 (0.20, 3.29) |
| Positive parenting | 95 | 0.48 (0.22, 1.03) | 96 | 0.66 (0.24, 1.80) | 94 | **0.34 (0.13, 0.93)** | 103 | 0.90 (0.25, 3.24) |
| Ethnicity Related Factors |  |  |  |  |  |  |  |  |
| Discrimination | 97 | 1.08 (1.01, 1.16) | 99 | 0.92 (0.76, 1.12) | 96 | 1.01 (0.92, 1.12) | 106 | 1.00 (0.88, 1.13) |
| Proactive coping | 100 | 0.86 (0.68, 1.08) | 101 | 0.76 (0.51, 1.14) | 98 | 0.86 (0.62, 1.19) | 108 | 1.13 (0.76, 1.68) |
| Ethnic Identity  exploration | 89 | 0.52 (0.19, 1.38) | 90 | 0.21 (0.04, 1.18) | 88 | **0.21 (0.05, 0.93)** | 97 | 0.36 (0.06, 2.20) |
| Ethnic Identity  affirmation | 93 | 0.53 (0.22, 1.30) | 96 | 0.36 (0.10, 1.22) | 92 | 0.41 (0.13, 1.35) | 102 | 0.45 (0.13, 1.52) |
| Ethnic Identity  resolution | 96 | 0.44 (0.18, 1.08) | 97 | 0.39 (0.11, 1.43) | 94 | **0.15 (0.04, 0.56)** | 104 | 0.70 (0.19, 2.54) |
| Racial/ethnic  socialization | 97 | **0.92 (0.87, 0.98)** | 100 | **0.86 (0.76, 0.97)** | 96 | **0.90 (0.83, 0.98)** | 106 | **0.80 (0.67, 0.96)** |
| *Notes.* Sample sizes within columns vary due to incomplete data in predictor variable. Bolded estimates represent significant at p<0.05.  ^a^Based on 101 dyads including 93 caregiver-youth dyads in agreement and 8 discordant dyads where caregiver endorse SI and youth denies.  ^b^Based on 103 dyads including 100 caregiver-youth dyads in agreement and 3 discordant dyads where caregiver endorse SI and youth denies.  ^c^Based on 100 dyads including 96 caregiver-youth dyads in agreement and 4 discordant dyads where caregiver endorse SI and youth denies.  ^d^Based on 117 dyads including 107 caregiver-youth dyads in agreement and 3 discordant dyads where caregiver endorse SI and youth denies. | | | | | | | | |
